# Short-term outcomes in robotic-assisted versus conventional laparoscopic surgery for rectal cancer: population-based study

**DOI:** 10.1093/bjsopen/zrag050

**Published:** 2026-05-28

**Authors:** Carl Mertens, Pamela Buchwald, Peter Matthiessen, Henrik Jutesten, Soran Gadan, Fredrik Jörgren

**Affiliations:** Department of Surgery, Helsingborg Hospital, Lund University, Helsingborg, Sweden; Department of Surgery, Skåne University Hospital, Malmö, Lund University, Malmö, Sweden; Department of Surgery, Örebro University Hospital, Örebro, Örebro University, Örebro, Sweden; Department of Surgery, Skåne University Hospital, Malmö, Lund University, Malmö, Sweden; Department of Surgery, Örebro University Hospital, Örebro, Örebro University, Örebro, Sweden; Department of Surgery, Helsingborg Hospital, Lund University, Helsingborg, Sweden

**Keywords:** circumferential resection margin, total mesorectal excision, postoperative complications, conversion to open surgery

## Abstract

**Background:**

This retrospective cohort study compared short-term outcomes between robotic-assisted and conventional laparoscopic surgery for rectal cancer using data from the Swedish Colorectal Cancer Registry.

**Method:**

All patients undergoing elective minimally invasive surgery for rectal cancer between 2014 and 2021 and registered in the Swedish Colorectal Cancer Registry were assessed for eligibility, with patients who underwent robotic-assisted and laparoscopic rectal cancer resection included in the study. The primary outcome was a positive circumferential resection margin (CRM+). Secondary outcomes included conversion to open surgery, total mesorectal excision (TME) specimen quality, and 30-day overall and surgical complications. Multivariable logistic regression analyses were performed.

**Results:**

Of 12 703 patients registered during the study period, 10 914 underwent abdominal resection; of these, 5874 were analysed in this study (3578 robotic-assisted; 2296 conventional laparoscopic surgery). There was no difference in CRM+ between the robotic-assisted and conventional laparoscopic surgery groups (6.5% *versus* 5.9%, respectively; *P* = 0.291). Conversion to open surgery was more frequent in the conventional laparoscopic surgery group (16.1% *versus* 9.1%; *P* < 0.001). In addition, 30-day surgical complications were more common in the robotic-assisted laparoscopic surgery group (21.5% *versus* 19.3%; *P* = 0.044), including a higher rate of anastomotic leakage (10.9% *versus* 7.4%; *P* = 0.001). In multivariable analysis, neither technique was an independent predictor of CRM+ (odds ratio (OR) 0.99; 95% confidence interval (c.i.) 0.75 to 1.30; *P* = 0.925). For secondary outcomes robotic-assisted laparoscopic surgery reduced the risk of conversion to open surgery (OR 0.51; 95% c.i. 0.41 to 0.63; *P* < 0.001), but resulted in fewer complete TME specimens (OR 0.66; 95% c.i. 0.52 to 0.83; *P* < 0.001).

**Conclusion:**

No short-term oncological advantage in terms of radial margin positivity was demonstrated between the two techniques. Findings regarding conversion rates, TME specimen quality, and anastomotic leakage warrant further investigation.

## Teaser

This population-based study compared short-term outcomes of robotic-assisted *versus* conventional laparoscopic surgery for rectal cancer using data from the Swedish Colorectal Cancer Registry (2014–2021). Robotic-assisted surgery was associated with a lower risk of conversion to open surgery but a lower rate of complete total mesorectal excision specimens, with no difference in circumferential resection margin positivity or postoperative complications. No short-term oncological advantage was demonstrated between the two techniques, and long-term outcomes are needed.

## Introduction

Total mesorectal excision (TME) is the standard surgical technique for rectal cancer in the mid- and lower rectum^1^. Despite randomized clinical trials (RCTs) failing to demonstrate the non-inferiority of conventional laparoscopic surgery for oncological surrogate markers such as circumferential resection margin positivity (CRM+) and the quality of the total mesorectal excision (TME) specimen,the shift from open to minimally invasive surgery (MIS) continues^2–5^. Initially, conventional laparoscopic surgery was introduced, followed in recent years by robotic-assisted laparoscopic surgery. Robotic-assisted laparoscopic surgery offers technological advantages that may overcome the challenges of conventional laparoscopy in rectal cancer surgery, potentially improving oncological surrogate outcomes. MIS offers short-term advantages over open surgery, including reduced postoperative pain, less blood loss, faster recovery, and shorter hospital stay^2–5^. However, reports on long-term outcomes for MIS *versus* open surgery are scarce^6,7^.

Most comparative data on the two MIS techniques are of low quality, derived from single-centre RCTs and cohorts. Two multicentre RCTs found no short-term benefits, whereas a third favoured robotic-assisted laparoscopic surgery^8–10^. Meta-analyses suggest a slight advantage of robotic-assisted laparoscopic surgery in reducing the risk of CRM+ and conversion rates, with comparable long-term outcomes^11–14^.

Long-term outcomes and population-based data are lacking. Whether robotic-assisted laparoscopic surgery improves outcomes over conventional laparoscopic surgery remains a topic of ongoing research and debate. The Swedish national treatment guidelines recommend MIS for rectal cancer^15^. Currently in Sweden, MIS is performed in over 80% of rectal cancer patients undergoing abdominal resection, with the proportion of robotic-assisted laparoscopic surgeries steadily increasing over the past decade^16^.

The primary aim of this study was to compare the risk of CRM+ between robotic-assisted and conventional laparoscopic surgery in a population-based national cohort. Secondary aims included comparing the risks of conversion to open surgery, TME specimen quality, and overall or surgical complications.

## Methods

This study was approved by the Swedish Ethical Review Authority (dnr 2023-01022-01) and was conducted in accordance with the Declaration of Helsinki.

### Swedish Colorectal Cancer Registry

Since 1995, nearly all patients in Sweden diagnosed with adenocarcinoma of the rectum have been registered (> 99%) in the Swedish Colorectal Cancer Registry (SCRCR). In the SCRCR, an adenocarcinoma with the lower edge within 15 cm of the anal verge, as measured by rigid sigmoidoscopy, is classified as rectal cancer. The SCRCR collects comprehensive data on patient and tumour clinical characteristics, diagnostics, treatments, histopathology, and postoperative morbidity within 30 days of surgery. Oncological outcomes, including local recurrence and distant metastases, are recorded at 3 and 5 years after initial registration. Dates of death are obtained from the Swedish Population Registry and are continuously updated. The SCRCR has a low proportion of missing data and has high internal and external validity^17–19^. To date, 55 120 patients with rectal cancer have been registered in the SCRCR. In 2014, robotic-assisted laparoscopic surgery was included as a registered variable in the SCRCR data set.

### Study population

This study was a retrospective analysis of prospectively registered data from the SCRCR, including patients with rectal cancer, tumour node metastasis (TNM) stages I–IV, who underwent elective abdominal MIS (anterior resection, abdominoperineal resection, or Hartmann’s procedure) between 1 January 2014 and 31 December 2021, with 30-day follow-up.

**Fig. 1:**
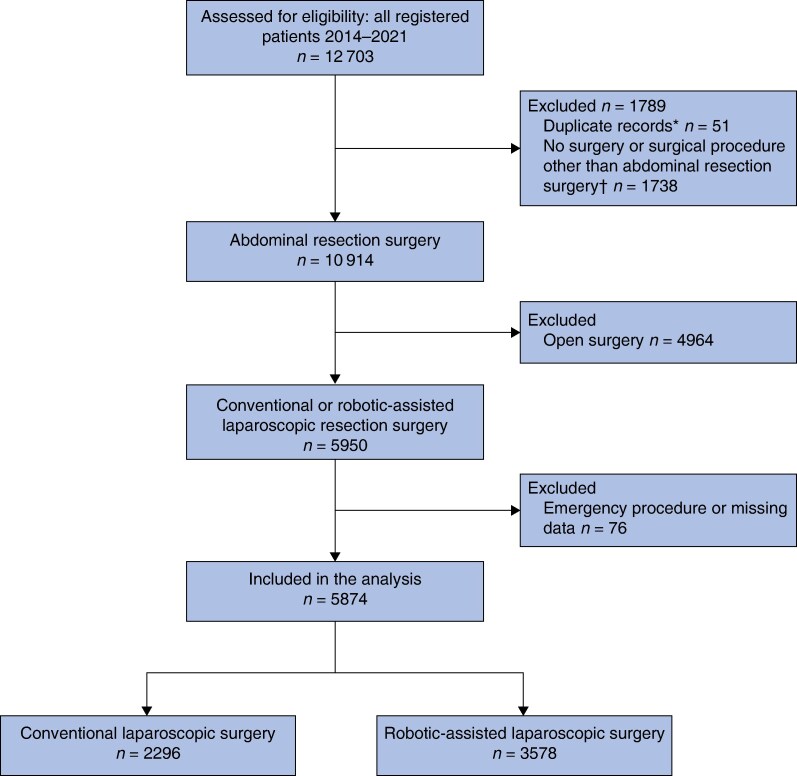
Study flow chart *Tumours rather than patients are registered in the Swedish colorectal cancer registry; hence, there may be duplicate reports made for a single patient with multiple tumours. In the event of duplicate records due to multiple synchronous rectal tumours, only the most advanced tumour was included in the analysis. †Abdominal resection surgery was defined as Hartmann’s procedure, anterior resection or abdominoperineal resection.

Initially, all patients registered in the SCRCR during the study period were assessed for eligibility. In the event of duplicate records due to multiple synchronous rectal tumours, only the most advanced tumour was included. Patients were excluded if they underwent procedures other than abdominal resection, open surgery, emergency surgery, or had missing key data. The final study cohort consisted of patients who underwent elective conventional laparoscopic or robotic-assisted laparoscopic rectal resection (*Fig. 1*).

### Outcomes of interest

The primary outcome of interest was CRM+, defined as tumour distance of ≤1 mm from the circumferential resection margin (CRM). Secondary outcomes included conversion to open surgery, specimen quality in TME, and 30-day overall and surgical complications. The macroscopic quality of the TME specimen was evaluated using Quirke’s method and categorized as complete (mesorectal plane), nearly complete (intramesorectal plane) and incomplete (muscularis propria plane)^20^. Surgical complications were defined according to the Clavien–Dindo classification. In the SCRCR, complications graded Clavien–Dindo II or higher are recorded. Surgical complications analysed in this study included wound infection, intra-abdominal infection, wound dehiscence, bleeding, anastomotic leakage, stoma-related complications, and other surgical complications as registered in the database.

### Statistical analysis

Patients were grouped based on MIS technique into robotic-assisted or conventional laparoscopic surgery groups. Categorical data are presented as absolute numbers with percentages and were compared using χ^2^ tests. Continuous data are reported as the median with interquartile range (i.q.r.) and were compared using Student’s *t* test. Statistical significance was set at *P* <0.05 (two-tailed). The impact of the MIS technique on outcomes was assessed using multivariable logistic regression analyses in two models. The first model was adjusted for age, sex, body mass index (BMI), clinical (c) TNM stage, American Society of Anesthesiologists fitness grade, tumour height, preoperative radiotherapy, preoperative chemotherapy, surgical procedure, intraoperative perforation, year of surgery, and TME specimen quality. The second model was adjusted for the same covariates except TME specimen quality. Missing data were excluded, because no imputation of data was performed. Adjusted odds ratios (ORs) with their 95% confidence interval (c.i.) were calculated in multivariable analyses, with ORs > 1.00 indicating worse outcomes for the test category compared with the reference category. All analyses were made on an intention-to-treat basis, and statistical significance was set at *P* < 0.05. SPSS® version 29.0 for Windows® (IBM, Armonk, NY, USA) and R® version 4.2.2 (R Foundation for Statistical Computing, Vienna, Austria) were used for data analyses.

## Results

During the study period, 12 703 patients were registered in the SCRCR, of whom 10 914 (85.9%) underwent abdominal resection surgery. Among these patients, 5950 (46.8%) underwent MIS. After applying the exclusion criteria, 5874 patients remained (2296 conventional laparoscopic surgery, 3578 robotic-assisted laparoscopic surgery) for analysis (*Fig. 1*). The proportion of patients undergoing robotic-assisted laparoscopic surgery within the MIS group significantly increased over the study period, from 43.0% in 2014 to 68.3% in 2021 (*Fig. S1*).

As indicated in *Table 1*, the robotic-assisted laparoscopic surgery group had a higher proportion of men and patients with more advanced tumours in terms of lower situated tumours, higher cT category, and more frequent radiological involvement of the CRM. Consequently, patients in the robotic-assisted laparoscopic surgery group were more often treated with chemoradiotherapy.

**Table 1:** Patient characteristics, treatment details, and tumour data for patients who underwent elective abdominal minimally invasive surgery for rectal cancer in Sweden, 2014–2021

	All patients (*n* = 5874)	Laparoscopic (*n* = 2296)	Robotic-assisted (*n* = 3578)	P*
Age (years), median (i.q.r.)	70.0 (62–76)	70.0 (62–77)	70.0 (61–76)	< 0.001
**ASA fitness grade**				0.354
I	988 (16.8%)	377 (16.4%)	611 (17.1%)	
II	3380 (57.5%)	1333 (58.1%)	2047 (57.2%)	
III	1358 (23.1%)	524 (22.8%)	834 (23.3%)	
IV	59 (1.0%)	26 (1.1%)	33 (0.9%)	
Missing	89 (1.6%)	36 (1.6%)	53 (1.5%)	
**Sex**				0.034
Male	3540 (60.3%)	1345 (58.6%)	2195 (61.3%)	
Female	2334 (39.7%)	951 (41.4%)	1383 (38.7%)	
**BMI† (kg/m^2^), median (i.q.r.)**	25.6 (23.3–28.4)	25.5 (23.1–28.4)	25.6 (23.4–28.4)	0.221
Missing	54 (0.9%)	22 (1.0%)	32 (0.9%)	
**Tumour height**‡				0.048
11–15 cm	1736 (29.6%)	720 (31.4%)	1016 (28.4%)	
6–10 cm	2492 (42.4%)	943 (41.1%)	1549 (43.3%)	
0–5 cm	1611 (27.4%)	615 (26.8%)	996 (27.8%)	
Missing	35 (0.6%)	18 (0.7%)	17 (0.5%)	
**cT category**				< 0.001
cT1-2	1916 (32.6%)	802 (34.9%)	1114 (31.1%)	
cT3	3140 (53.5%)	1236 (53.9%)	1904 (53.2%)	
cT4	677 (11.5%)	195 (8.5%)	482 (13.5%)	
cTx	133 (2.3%)	58 (2.5%)	75 (2.1%)	
Missing	8 (0.1%)	5 (0.2%)	3 (0.1%)	
**cN category**				0.202
cN0	2656 (45.2%)	1049 (45.7%)	1607 (44.9%)	
cN1-2	3140 (53.5	1222 (53.2%)	1918 (53.6%)	
cNx	72 (1.2%)	21 (0.9%)	51 (1.4%)	
Missing	6 (0.1%)	4 (0.2%)	2 (0.1%)	
**cM category**				0.448
cM0	5528 (94.1%)	2166 (94.4%)	3362 (94.0%)	
cM1	329 (5.6%)	122 (5.3%)	207 (5.8%)	
Missing	17 (0.3%)	8 (0.3%)	9 (0.2%)	
**cTNM**				0.381
Stage I	1125 (19.2%)	424 (18.5%)	701 (19.6%)	
Stage II	976 (16.6%)	365 (15.9%)	611 (17.1%)	
Stage III	1697 (28.9%)	633 (27.6%)	1064 (29.7%)	
Stage IV	304 (5.2%)	99 (4.3%)	205 (5.7%)	
Missing	1772 (30.1%)	775 (33.7%)	997 (27.9%)	
**cCRM+**				< 0.001
Yes	696 (11.8%)	191 (8.3%)	505 (14.1%)	
No	3604 (61.4%)	1365 (59.5%)	2239 (62.6%)	
Missing	1574 (26.8%)	740 (32.2%)	834 (23.3%)	
**Preoperative radiotherapy**				0.348
Yes	3300 (56.2%)	1272 (55.4%)	2028 (56.7%)	
No	2566 (43.7%)	1020 (44.4%)	1546 (43.2%)	
Missing	8 (0.1%)	4 (0.2%)	4 (0.1%)	
**Preoperative chemotherapy**				< 0.001
Yes	1007 (17.1%)	252 (11.0%)	755 (21.1%)	
No	4858 (82.7%)	2040 (88.9%)	2818 (78.8%)	
Missing	9 (0.2%)	4 (0.2%)	5 (0.1%)	
**Surgical procedure**				0.052
Hartmann's procedure	638 (10.9%)	269 (11.7%)	369 (10.3%)	
Anterior resection	3065 (52.5%)	1156 (50.3%)	1909 (53.4%)	
Abdominoperineal resection	2171 (37.0%)	871 (37.9%)	1300 (36.3%)	
**Conversion to open surgery**				< 0.001
Yes	696 (11.9%)	370 (16.1%)	326 (9.1%)	
No	5171 (88.0%)	1924 (83.8%)	3247 (90.7%)	
Missing	7 (0.1%)	2 (0.1%)	5 (0.1%)	
**Intraoperative rectal washout**§				
Yes	3349 (90.5%)	1276 (89.6%)	2073 (91.0%)	0.543
No	283 (7.6%)	113 (7.9%)	170 (7.5%)	
Missing	71 (1.9%)	36 (2.5%)	35 (1.5%)	
**Intraoperative bowel perforation**				
All	219 (3.7%)	78 (3.4%)	141 (3.9%)	0.282
Tumour adjacent	113 (1.9%)	36 (1.5%)	77 (2.2%)	0.464
Other location	97 (1.7%)	38 (1.7%)	59 (1.6%)	
Not specified	9 (0.1%)	4 (0.2%)	5 (0.1%)	
**Intraoperative bleeding (ml), median (i.q.r.)**	100 (50–200)	75 (25–200)	100 (50–200)	0.060
Missing	104 (1.8%)	43 (1.9%)	61 (1.7%)	
**Surgeon competence**				0.164
Colorectal surgeon	5812 (98.9%)	2277 (99.2%)	3535 (98.8%)	
General surgeon	17 (0.3%)	9 (0.4%)	8 (0.2%)	
Missing	45 (0.8%)	10 (0.4%)	35 (1.0%)	
**Operative time (min), median (i.q.r.)**	338 (261–415)	314 (238–397)	351 (276–424)	< 0.001
Missing	28 (0.5%)	11 (0.5%)	18 (0.5%)	
**LOS# (days), median (i.q.r.)**	7 (5–10)	7 (5–10)	6 (4–10)	0.262
Missing	86 (1.5%)	30 (1.3%)	56 (1.6%)	

Values are *n* (%) unless otherwise stated. †Patients with BMI values <15 or >50 kg/m^2^ were excluded because they were few in number and likely not accurately recorded. ‡Tumour height was determined as the distance from the anal verge. §For anterior resection and Hartmann’s procedure. #The LOS was calculated by subtracting the date of surgery from the date of discharge. Patients with an LOS <1 or >180 days were excluded because they were few in number and likely not accurately recorded. i.q.r., interquartile range; ASA, American Society of Anesthesiologists; BMI, body mass index;, clinical; TNM, tumour node metastasis staging system; CRM+, positive circumferential resection margin; LOS, length of hospital stay. *χ² test or Student’s t-test.

### Surgical outcomes

In the study population, conversion to open surgery occurred in 696 patients (11.9%) overall. The conversion rate was lower in the robotic-assisted than conventional laparoscopic surgery group (9.1% *versus* 16.1%; *P* < 0.001; *Table 1*). The overall conversion rate decreased over time, but the difference between the two MIS techniques, favouring robotic-assisted laparoscopic surgery, persisted (*Fig. S2*). Operative time was shorter in the conventional than robotic-assisted laparoscopic surgery group (median 314 (i.q.r. 238–397) *versus* 351 (i.q.r. 276–242) min, respectively; *P* < 0.001). In the case of anterior resection, a temporary stoma was fashioned at the time of the index surgery in 72.2% of patients, with no difference between the conventional laparoscopic and robotic-assisted surgery groups (72.4% *versus* 72.1%, respectively; *P* = 0.906).

### Pathological outcomes

Overall, 366 patients (6.2%) had a CRM+, with no difference between the robotic-assisted and conventional laparoscopic surgery groups (6.5% *versus* 5.9%, respectively; *P* = 0.291). However, complete TME specimens were less frequently observed in the robotic-assisted laparoscopic surgery group. The number of harvested lymph nodes was higher in the robotic-assisted laparoscopic surgery group. The mean distal resection margin was longer in the conventional than robotic-assisted laparoscopic surgery group (37.22 *versus* 35.77 mm; *P* = 0.035), whereas the median distal resection margin did not differ between the two groups (*Table 2*).

**Table 2:** Pathology outcomes data for patients who underwent elective abdominal minimally invasive surgery for rectal cancer in Sweden, 2014–2021

	All patients (*n* = 5874)	Laparoscopic (*n* = 2296)	Robotic-assisted (*n* = 3578)	P*
**(y)pT category†**				< 0.001
(y)pT0	193 (3.3%)	54 (2.4%)	139 (3.9%)	
(y)pT1	568 (9.7%)	234 (10.2%)	334 (9.3%)	
(y)pT2	1803 (30.7%)	675 (29.4%)	1128 (31.5%)	
(y)pT3	2888 (49.2%)	1188 (51.7%)	1700 (47.5%)	
(y)pT4	244 (4.1%)	86 (3.7%)	158 (4.4%)	
(y)pTx or missing	178 (3.0%)	59 (2.5%)	119 (3.4%)	
**(y)pN category†**				0.084
(y)pN0	3564 (60.7%)	1409 (61.4%)	2155 (60.2%)	
(y)pN1	1602 (27.3%)	595 (25.9%)	1007 (28.1%)	
(y)pN2	539 (9.2%)	232 (10.1%)	307 (8.6%)	
(y)pNx or missing	169 (2.8%)	60 (2.6%)	109 (3.0%)	
**Harvested lymph nodes (*n*), median (i.q.r.)**	19 (14–26)	18 (14–24)	20 (14–28)	< 0.001
Missing data	160 (2.7%)	59 (2.6%)	101 (2.8%)	
**Distal resection margin‡ (mm), median (i.q.r.)**	35 (20–50)	35 (20–50)	35 (20–50)	0.035
Missing data	1638 (27.9%)	662 (28.8%)	976 (27.3%)	
**CRM**				0.291
Positive (≤1 mm)	366 (6.2%)	135 (5.9%)	231 (6.5%)	
Negative (>1 mm)	4970 (84.6%)	1972 (85.9%)	2998 (83.8%)	
Missing	538 (9.2%)	189 (8.2%)	349 (9.8%)	
**Macroscopic quality of TME specimen**				< 0.001
Complete	2329 (39.6%)	819 (35.7%)	1510 (42.2%)	
Nearly complete	598 (10.2%)	140 (6.1%)	458 (12.8%)	
Incomplete	236 (4.0%)	62 (2.7%)	174 (4.9%)	
Missing	2711 (46.2%)	1275 (55.6%)	1436 (40.1%)	

Values are *n* (%) unless otherwise stated. †TNM stage based on pathology reports. ‡Patients with a distal resection margin >150 mm were excluded because they were few (15) and likely not accurately recorded given that the study only included rectal tumours. y, post-therapy TNM; p, pathological TNM; i.q.r., interquartile range; CRM, circumferential resection margin; TME, total mesorectal excision; TNM, tumour node metastasis staging system. *χ² or Student’s t-test.

### Complications

Postoperative complications were recorded in 2163 patients (36.8%). No difference was found in overall postoperative complications between the two groups, but surgical complications were more frequent in the robotic-assisted than conventional laparoscopic surgery group (21.5% *versus* 19.3%; *P* = 0.044; *Table 3*). Subgroup analysis revealed higher rates of wound infection in the conventional than robotic-assisted laparoscopic surgery group (5.8% *versus* 4.4%; *P* = 0.020) and higher rates of anastomotic leakage in the robotic-assisted than conventional laparoscopic surgery group (10.9% *versus* 7.4%; *P* = 0.001).

**Table 3:** Complication data for patients who underwent elective abdominal minimally invasive surgery for rectal cancer in Sweden, 2014–2021

	All patients (*n* = 5874)	Laparoscopic (*n* = 2296)	Robotic-assisted (*n* = 3578)	P*
Overall complications	2163 (36.8%)	844 (36.8%)	1319 (36.9%)	0.919
**Surgical complications**				
Overall surgical complications	1214 (20.7%)	444 (19.3%)	770 (21.5%)	0.044
Intra-abdominal bleeding	52 (0.9%)	23 (1.0%)	29 (0.8%)	0.445
Wound infection	292 (5.0%)	133 (5.8%)	159 (4.4%)	0.020
Wound dehiscence	51 (0.9%)	24 (1.0%)	27 (0.8%)	0.241
Intra-abdominal infection	286 (4.9%)	100 (4.4%)	186 (5.2%)	0.143
Anastomotic leak†	295 (9.6%)	86 (7.4%)	209 (10.9%)	0.001
Reoperation	548 (9.3%)	197 (8.6%)	351 (9.8%)	0.111
Need for ICU admission	179 (3.0%)	77 (3.4%)	102 (2.9%)	0.278
Death within 30 days	31 (0.5%)	14 (0.6%)	17 (0.5%)	0.487
**Non-surgical complications**				
Infectious	375 (6.4%)	144 (6.3%)	231 (6.5%)	0.778
Cardiovascular	123 (2.1%)	44 (1.9%)	79 (2.2%)	0.446
Neurological	27 (0.5%)	7 (0.3%)	20 (0.6%)	0.160

Values are *n* (%). †For anterior resection. ICU, intensive care unit. *χ² test or Student’s t-test.

### Multivariable analysis

Multivariable logistic regression analysis, adjusted for covariates, was conducted to identify whether any of the MIS techniques was an independent risk factor for CRM+, conversion to open surgery, quality of the TME specimen, postoperative complications, and surgical complications in two models (*Table 4* and *Table S1*). In the first model, robotic-assisted laparoscopic surgery reduced the risk of conversion to open surgery (adjusted OR 0.49; 95% c.i. 0.36 to 0.67; *P* < 0.001), but resulted in fewer complete TME specimens (adjusted OR 0.66; 95% c.i. 0.52 to 0.83; *P* < 0.001) than conventional laparoscopy (*Table 4*). For the other outcomes analysed, no differences were found between the two MIS techniques. In the second model, the analysis yielded the same results as in the first model (*Table S1*).

**Table 4:** Multivariable regression analysis of the impact of minimally invasive surgery techniques on short-term outcomes data for patients who underwent elective abdominal minimally invasive surgery for rectal cancer in Sweden, 2014–2021

Outcome	Surgical technique	No. of observations	Multivariable analysis*	
			Odds ratio	*P*
CRM+		1938		0.099
	Laparoscopic		1.0	
	Robotic-assisted		0.69 (0.44, 1.06)	
Conversion to open surgery		2050		< 0.001
	Laparoscopic		1.0	
	Robotic-assisted		0.49 (0.36, 0.67)	
Complete TME specimen		2052		< 0.001
	Laparoscopic		1.0	
	Robotic-assisted		0.66 (0.52, 0.83)	
Overall complications		2044		0.078
	Laparoscopic		1.0	
	Robotic-assisted		0.83 (0.68, 1.02)	
Surgical complications		2052		0.824
	Laparoscopic		1.0	
	Robotic-assisted		0.97 (0.76, 1.24)	

Values in parentheses are 95% confidence intervals. *Adjusted for age, sex, body mass index, tumour node metastasis stage, American Society of Anesthesiologists fitness grade, tumour height, preoperative radiotherapy, preoperative chemotherapy, surgical procedure, intraoperative perforation, year of surgery, and TME specimen quality. CRM+, positive circumferential resection margin; TME, total mesorectal excision.

Additional multivariable analyses conducted within subgroups defined by sex, cT category, neoadjuvant treatment, and time period yielded results consistent with the main analyses (*Tables S2–S7* and *Fig. S2*). Robotic-assisted laparoscopic surgery remained associated with lower conversion rates, but also with fewer complete TME specimens across all major subgroups, including male sex, cT3–T4 tumours, and patients receiving preoperative radiotherapy. No significant differences were observed in CRM+. Among patients who received combined radiotherapy and chemotherapy, subgroup numbers were small, and no significant differences were detected.

## Discussion

This population-based study, using data from the SCRCR, compared short-term outcomes of robotic-assisted and conventional laparoscopic surgery for rectal cancer. Neither of the techniques proved superior.

The primary outcome, CRM+, did not differ between the robotic-assisted and conventional laparoscopic surgery groups, aligning with previous studies^9,10,12^. However, a recent large RCT reported improved CRM outcomes with robotic-assisted laparoscopic surgery^8^. An adequate CRM is critical for optimal oncological outcomes, because CRM+ (≤ 1 mm) is strongly associated with an increased risk of local recurrence and distant metastasis^21,22^. The present findings suggest comparable oncological outcomes between the two approaches.

The robotic-assisted laparoscopic surgery group had a lower conversion rate to open surgery. In multivariable analysis, robotic-assisted laparoscopic surgery emerged as an independent factor for reduced conversion risk. In contrast, the COLRAR^10^ and ROLARR^9^ trials did not observe any difference in conversion rates between the two MIS techniques. However, in subgroup analyses of the ROLARR trial^9^, lower conversion rates were found in men and patients with obesity in the robotic-assisted laparoscopic surgery group, whereas the REAL trial^8^ reported notably lower conversion rates overall in the robotic-assisted laparoscopic surgery group. In addition, several systematic reviews have demonstrated reduced conversion rates for the robotic-assisted laparoscopic surgery group^11,12,14^. The higher overall conversion rate in the present study compared with other studies may reflect surgeons’ learning curves with robotic-assisted laparoscopic surgery and the higher BMI among patients in the present study than in the two RCTs^8,10^ of Asian patients.

The robotic-assisted laparoscopic surgery group had a higher rate of incomplete or nearly incomplete TME specimens and was inferior to conventional laparoscopic surgery in the multivariable analysis. This contrasts with previous trials (ROLARR^9^ and COLRAR^10^), which reported comparable outcomes, and the REAL trial^8^, which favoured robotic-assisted laparoscopic surgery. Because an incomplete TME specimen is considered an indicator of poorer long-term oncological outcomes^20,23,24^, this finding is of importance. However, it is essential to acknowledge the substantial amount of missing data for this variable, which limits the strength of the conclusions of this study. Reporting of TME quality in the SCRCR is not mandatory, which likely contributes to the high proportion of missing data. Notably, the proportion of missing data has remained stable throughout the study period, and continued missing data is seen for more recent years. Pathology reporting in Sweden is otherwise standardized, and Swedish pathologists are well trained in specimen assessment according to Quirke, following nationwide workshops during the implementation of TME surgery. Thus, although the missing data are unlikely to be random, the exact mechanism cannot be determined, and this limitation should be kept in mind when interpreting the results. Poor TME quality has been associated with an increased risk of local recurrence^24^. Because local recurrence is captured in the long-term follow-up data within the SCRCR, future analyses may help clarify whether the differences observed in TME quality translate into clinically relevant oncological outcomes.

The reported overall complication rate in the present cohort was higher than in the published RCTs^8–10^. Multivariable analysis showed no increased risk of overall surgical complications in either group. This finding deviates from the REAL trial^8^ and the systematic review by Wang *et al*.^14^, in which fewer complications were reported for the robotic-assisted laparoscopic group, whereas other publications^9,10,12^ have found no differences. However, in the present study, among specific surgical complications, wound infections were more common in the conventional laparoscopic surgery group and anastomotic leakages were more common in the robotic-assisted laparoscopic surgery group. These findings are in contrast with previous reports, in which no differences were reported between the MIS techniques for these complications^8–12,14^ .

Robotic-assisted laparoscopic surgery would, in theory, offer technical advantages over conventional laparoscopic surgery in rectal cancer, particularly in deep, narrow pelvises of patients with obesity, due to superior three-dimensional visualization, enhanced instrument articulation, and greater stability. This should improve short- and long-term oncological outcomes, yet the evidence remains weak and contradictory. Two RCTs (ROLARR^9^ and COLRAR^10^) found no short-term benefits, whereas REAL^8^ supported robotic-assisted laparoscopic surgery. However, ROLARR^9^ may have been underpowered for the primary outcome (conversion rates) and COLRAR^10^ was terminated prematurely due to poor accrual of data, casting doubt on the conclusions of the study. The generalizability of Asian RCTs with regard to Western populations is also questionable, given BMI-related surgical complexity^8,10^. Moreover, most systematic reviews rely on small single-centre RCTs, retrospective case-control series, or observational data, raising concerns about their reliability.

The SCRCR is a national population-based registry with a large, valid cohort that reflects routine clinical care, in which prospective data registration enables multivariable analysis and adjustment for confounders despite the retrospective design^17–19^. A potential limitation is that the surgeons performing the robotic-assisted technique were in the early learning phase. In contrast, the surgeons performing conventional laparoscopic surgery were more experienced, because the study period started shortly after the introduction of robotic-assisted laparoscopic rectal cancer surgery in Sweden. Adjustment was made for the year of surgery in the multivariable analysis to overcome this confounder to some extent. However, the registry does not include information on when individual centres introduced robotic surgery, and there were likely differences among centres in baseline experience with laparoscopic rectal surgery. This variation may have influenced outcomes to some degree. Future studies linking registry data with hospital- or surgeon-level information could help clarify the impact of learning curves.

To further explore these factors, subgroup analyses were performed. These results were consistent with the main findings of this study, with robotic-assisted laparoscopic surgery consistently associated with a lower risk of conversion and a lower proportion of complete TME specimens across subgroups defined by sex, tumour stage, neoadjuvant therapy, and time period. The consistency of these results across analyses supports the reliability of the findings, although differences in TME quality should be interpreted with caution due to missing data.

Selection bias may also have influenced outcomes because the robotic-assisted laparoscopic surgery group consisted of patients with more advanced, with proportionally more men, lower situated tumours, higher cT categories, more cCRM+ tumours, and more patients receiving neoadjuvant therapy. In addition, the high proportion of missing data for TME specimen quality may have affected the analyses. Multiple imputation of the variable was not appropriate, because analysis of the data set revealed a higher proportion of missing data in some areas of Sweden; thus, the missing data could not be considered missing at random. To address this limitation, multivariable analysis was performed in two models, with and without adjustment for TME specimen quality, yielding identical results.

This study highlights that although robotic-assisted laparoscopic surgery offers certain technical benefits, such as reduced conversion rates, it does not consistently outperform conventional laparoscopy across all measures. Given the global and national rise in robotic-assisted laparoscopic surgery procedures, further research is needed. Future studies should focus on long-term oncological outcomes and complications. The impact of conversion to open surgery on long-term outcomes and the incidence of anastomotic leakage are two central issues that warrant special emphasis.

## Supplementary Material

zrag050_Supplementary_Data

## Data Availability

The study data can be provided upon reasonable request to the corresponding author.
