# Supplementary material for: Short-term outcomes in robotic-assisted versus conventional laparoscopic surgery for rectal cancer: population-based study
Source: BJS Open. 2026 May 28;10(3):zrag050. doi: 10.1093/bjsopen/zrag050 (PMC13217427; doi:10.1093/bjsopen/zrag050)
Supplement: zrag050_Supplementary_Data [file zrag050_supplementary_data.docx]

**Comparison of short-term outcomes in robotic-assisted laparoscopic surgery *versus* conventional laparoscopic surgery for rectal cancer - a population-based study**

Carl Mertens (C.M.) MD^1^, Pamela Buchwald (P.B.) MD, PhD^2^, Peter Matthiessen (P.M.) MD, PhD^3^, Henrik Jutesten (H.J.) MD, PhD^2^, Soran Gadan (S.G.) MD, PhD^3^, Fredrik Jörgren (F.J.) MD, PhD^1^

^1^Department of Surgery, Helsingborg Hospital, Lund University, Helsingborg, Sweden

^2^Department of Surgery, Skåne University Hospital, Malmö, Lund University, Malmö, Sweden

^3^ Department of Surgery, Örebro University Hospital, Örebro, Örebro University, Örebro, Sweden

##### **Correspondence**

Carl Mertens MD, Department of Surgery, Helsingborg Hospital, Charlotte Yhlens gata 10, SE-254 37 Helsingborg, Sweden

Email: carl.mertens@med.lu.se

Telephone: +46-(0) 42-4061000

Fax: +46-(0) 42-4061516

**Supplementary Materials - Index**

| **Supplementary Figures and Tables** |  |
| --- | --- |
| Table S1. | *pag. 2* |
| Table S2. | *pag. 3* |
| Table S3. | *pag. 4* |
| Table S4. | *pag. 5* |
| Table S5. | *pag. 6* |
| Table S6. | *pag. 7* |
| Table S7. | *pag. 8* |
| Fig. S1 | *pag. 9* |
| Fig. S2 | *pag. 10* |

**Supplementary Figures and Tables**

| **Table S1. Multivariable regression analysis of impact of minimally invasive surgery techniques on short-term outcomes data for patients who had elective abdominal minimally invasive surgery for rectal cancer in Sweden, 2014-2021** | | | | |
| --- | --- | --- | --- | --- |
| **Outcome** | **Surgical technique** | **Number**  **of observations** | **Multivariable analysis*** | |
|  |  |  | **Odds ratio** | ***P*** |
| CRM+ |  | 3624 |  | 0.925 |
|  | Laparoscopic |  | 1.0 |  |
|  | Robotic-assisted |  | 0.99 (0.75, 1.30) |  |
| Conversion to open surgery |  | 3965 |  | <0.001 |
|  | Laparoscopic |  | 1.0 |  |
|  | Robotic-assisted |  | 0.51 (0.41, 0.63) |  |
| Complete TME specimen |  | 2052 |  | <0.001 |
|  | Laparoscopic |  | 1.0 |  |
|  | Robotic-assisted |  | 0.66 (0.52, 0.83) |  |
| Overall complications |  | 3965 |  | 0.739 |
|  | Laparoscopic |  | 1.0 |  |
|  | Robotic-assisted |  | 0.98 (0.85, 1.12) |  |
| Surgical complications |  | 3970 |  | 0.177 |
|  | Laparoscopic |  | 1.0 |  |
|  | Robotic-assisted |  | 1.12 (0.95, 1.33) |  |

Values in parentheses are 95 per cent confidence intervals. CRM*,* Circumferential Resection Margin; TME. Total Mesorectal Excision. *Adjusted for age, sex, BMI, TNM stage, ASA fitness grade, tumour height, preoperative radiotherapy, preoperative chemotherapy, surgical procedure, intraoperative perforation and year of surgery.

| **Table S2. Multivariable regression analysis of impact of minimally invasive surgery techniques on short-term outcomes data for male patients who had elective abdominal minimally invasive surgery for rectal cancer in Sweden, 2014–2021.** | | | | |
| --- | --- | --- | --- | --- |
| **Outcome** | **Surgical technique** | **Number**  **of observations** | **Multivariable analysis*** | |
|  |  |  | **Odds ratio** | ***P*** |
| CRM+ |  | 1213 |  | 0.132 |
|  | Laparoscopic |  | 1.0 |  |
|  | Robotic-assisted |  | 0.65 (0.36, 1.13) |  |
| Conversion to open surgery |  | 1273 |  | <0.001 |
|  | Laparoscopic |  | 1.0 |  |
|  | Robotic-assisted |  | 0.44 (0.30, 0.64) |  |
| Complete TME specimen |  | 1275 |  | 0.006 |
|  | Laparoscopic |  | 1.0 |  |
|  | Robotic-assisted |  | 0.67 (0.50, 0.89) |  |
| Overall complications |  | 1269 |  | 0.129 |
|  | Laparoscopic |  | 1.0 |  |
|  | Robotic-assisted |  | 0.82 (0.63, 1.06) |  |
| Surgical complications |  | 1275 |  | 0.716 |
|  | Laparoscopic |  | 1.0 |  |
|  | Robotic-assisted |  | 0.95 (0.70, 1.28) |  |

Values in parentheses are 95 per cent confidence intervals. CRM*,* Circumferential Resection Margin; TME. Total Mesorectal Excision. *Adjusted for age, sex, BMI, TNM stage, ASA fitness grade, tumour height, preoperative radiotherapy, preoperative chemotherapy, surgical procedure, intraoperative perforation, year of surgery, and TME specimen quality.

| **Table S3. Multivariable regression analysis of impact of minimally invasive surgery techniques on short-term outcomes data for patients with cT3–T4 tumours who had elective abdominal minimally invasive surgery for rectal cancer in Sweden, 2014–2021.** | | | | |
| --- | --- | --- | --- | --- |
| **Outcome** | **Surgical technique** | **Number**  **of observations** | **Multivariable analysis*** | |
|  |  |  | **Odds ratio** | ***P*** |
| CRM+ |  | 1086 |  | 0.192 |
|  | Laparoscopic |  | 1.0 |  |
|  | Robotic-assisted |  | 0.72 (0.43, 1.18) |  |
| Conversion to open surgery |  | 1106 |  | <0.001 |
|  | Laparoscopic |  | 1.0 |  |
|  | Robotic-assisted |  | 0.40 (0.26, 0.61) |  |
| Complete TME specimen |  | 1107 |  | <0.001 |
|  | Laparoscopic |  | 1.0 |  |
|  | Robotic-assisted |  | 0.59 (0.43, 0.80) |  |
| Overall complications |  | 1103 |  | 0.061 |
|  | Laparoscopic |  | 1.0 |  |
|  | Robotic-assisted |  | 0.77 (0.59, 1.01) |  |
| Surgical complications |  | 1107 |  | 0.534 |
|  | Laparoscopic |  | 1.0 |  |
|  | Robotic-assisted |  | 0.90 (0.66, 1.25) |  |

Values in parentheses are 95 per cent confidence intervals. CRM*,* Circumferential Resection Margin; TME. Total Mesorectal Excision. *Adjusted for age, sex, BMI, TNM stage, ASA fitness grade, tumour height, preoperative radiotherapy, preoperative chemotherapy, surgical procedure, intraoperative perforation, year of surgery, and TME specimen quality.

| **Table S4. Multivariable regression analysis of impact of minimally invasive surgery techniques on short-term outcomes data for patients who received preoperative radiotherapy who had elective abdominal minimally invasive surgery for rectal cancer in Sweden, 2014–2021.** | | | | |
| --- | --- | --- | --- | --- |
| **Outcome** | **Surgical technique** | **Number**  **of observations** | **Multivariable analysis*** | |
|  |  |  | **Odds ratio** | ***P*** |
| CRM+ |  | 1047 |  | 0.171 |
|  | Laparoscopic |  | 1.0 |  |
|  | Robotic-assisted |  | 0.70 (0.41, 1.16) |  |
| Conversion to open surgery |  | 1132 |  | 0.006 |
|  | Laparoscopic |  | 1.0 |  |
|  | Robotic-assisted |  | 0.55 (0.36, 0.84) |  |
| Complete TME specimen |  | 1133 |  | 0.031 |
|  | Laparoscopic |  | 1.0 |  |
|  | Robotic-assisted |  | 0.72 (0.53, 0.97) |  |
| Overall complications |  | 1128 |  | 0.037 |
|  | Laparoscopic |  | 1.0 |  |
|  | Robotic-assisted |  | 0.75 (0.57, 0.98) |  |
| Surgical complications |  | 1133 |  | 0.544 |
|  | Laparoscopic |  | 1.0 |  |
|  | Robotic-assisted |  | 0.91 (0.67, 1.24) |  |

Values in parentheses are 95 per cent confidence intervals. CRM*,* Circumferential Resection Margin; TME. Total Mesorectal Excision. *Adjusted for age, sex, BMI, TNM stage, ASA fitness grade, tumour height, preoperative radiotherapy, preoperative chemotherapy, surgical procedure, intraoperative perforation, year of surgery, and TME specimen quality.

| **Table S5. Multivariable regression analysis of impact of minimally invasive surgery techniques on short-term outcomes data for patients who received preoperative radio- and chemotherapy who had elective abdominal minimally invasive surgery for rectal cancer in Sweden, 2014–2021.** | | | | |
| --- | --- | --- | --- | --- |
| **Outcome** | **Surgical technique** | **Number**  **of observations** | **Multivariable analysis*** | |
|  |  |  | **Odds ratio** | ***P*** |
| CRM+ |  | 320 |  | 0.376 |
|  | Laparoscopic |  | 1.0 |  |
|  | Robotic-assisted |  | 0.63 (0.21, 1.71) |  |
| Conversion to open surgery |  | 372 |  | 0.827 |
|  | Laparoscopic |  | 1.0 |  |
|  | Robotic-assisted |  | 1.12 (0.43, 3.35) |  |
| Complete TME specimen |  | 372 |  | 0.358 |
|  | Laparoscopic |  | 1.0 |  |
|  | Robotic-assisted |  | 0.74 (0.38, 1.39) |  |
| Overall complications |  | 372 |  | 0.429 |
|  | Laparoscopic |  | 1.0 |  |
|  | Robotic-assisted |  | 0.80 (0.46, 1.39) |  |
| Surgical complications |  | 372 |  | 0.921 |
|  | Laparoscopic |  | 1.0 |  |
|  | Robotic-assisted |  | 1.03 (0.55, 2.03) |  |

Values in parentheses are 95 per cent confidence intervals. CRM*,* Circumferential Resection Margin; TME. Total Mesorectal Excision. *Adjusted for age, sex, BMI, TNM stage, ASA fitness grade, tumour height, preoperative radiotherapy, preoperative chemotherapy, surgical procedure, intraoperative perforation, year of surgery, and TME specimen quality.

| **Table S6. Multivariable regression analysis of impact of minimally invasive surgery techniques on short-term outcomes data for patients who had elective abdominal minimally invasive surgery for rectal cancer in Sweden, 2014-2017** | | | | |
| --- | --- | --- | --- | --- |
| **Outcome** | **Surgical technique** | **Number**  **of observations** | **Multivariable analysis*** | |
|  |  |  | **Odds ratio** | ***P*** |
| CRM+ |  | 262 |  | 0.354 |
|  | Laparoscopic |  | 1.0 |  |
|  | Robotic-assisted |  | 0.55 (0.14, 1.90) |  |
| Conversion to open surgery |  | 284 |  | 0.009 |
|  | Laparoscopic |  | 1.0 |  |
|  | Robotic-assisted |  | 0.29 (0.11, 0.74) |  |
| Complete TME specimen |  | 284 |  | 0.030 |
|  | Laparoscopic |  | 1.0 |  |
|  | Robotic-assisted |  | 0.53 (0.29, 0.94) |  |
| Overall complications |  | 283 |  | 0.163 |
|  | Laparoscopic |  | 1.0 |  |
|  | Robotic-assisted |  | 0.68 (0.39, 1.17) |  |
| Surgical complications |  | 284 |  | 0.929 |
|  | Laparoscopic |  | 1.0 |  |
|  | Robotic-assisted |  | 1.03 (0.51, 2.13) |  |

Values in parentheses are 95 per cent confidence intervals. CRM*,* Circumferential Resection Margin; TME. Total Mesorectal Excision. *Adjusted for age, sex, BMI, TNM stage, ASA fitness grade, tumour height, preoperative radiotherapy, preoperative chemotherapy, surgical procedure, intraoperative perforation, year of surgery, and TME specimen quality.

| **Table S7. Multivariable regression analysis of impact of minimally invasive surgery techniques on short-term outcomes data for patients who had elective abdominal minimally invasive surgery for rectal cancer in Sweden, 2018-2021** | | | | |
| --- | --- | --- | --- | --- |
| **Outcome** | **Surgical technique** | **Number**  **of observations** | **Multivariable analysis*** | |
|  |  |  | **Odds ratio** | ***P*** |
| CRM+ |  | 1676 |  | 0.096 |
|  | Laparoscopic |  | 1.0 |  |
|  | Robotic-assisted |  | 0.67 (0.41, 1.07) |  |
| Conversion to open surgery |  | 1766 |  | <0.001 |
|  | Laparoscopic |  | 1.0 |  |
|  | Robotic-assisted |  | 0.48 (0.34, 0.68) |  |
| Complete TME specimen |  | 1768 |  | 0.005 |
|  | Laparoscopic |  | 1.0 |  |
|  | Robotic-assisted |  | 0.70 (0.55, 0.90) |  |
| Overall complications |  | 1761 |  | 0.216 |
|  | Laparoscopic |  | 1.0 |  |
|  | Robotic-assisted |  | 0.87 (0.70, 1.08) |  |
| Surgical complications |  | 1768 |  | 0.816 |
|  | Laparoscopic |  | 1.0 |  |
|  | Robotic-assisted |  | 0.97 (0.75, 1.26) |  |

Values in parentheses are 95 per cent confidence intervals. CRM*,* Circumferential Resection Margin; TME. Total Mesorectal Excision. *Adjusted for age, sex, BMI, TNM stage, ASA fitness grade, tumour height, preoperative radiotherapy, preoperative chemotherapy, surgical procedure, intraoperative perforation and year of surgery.

**
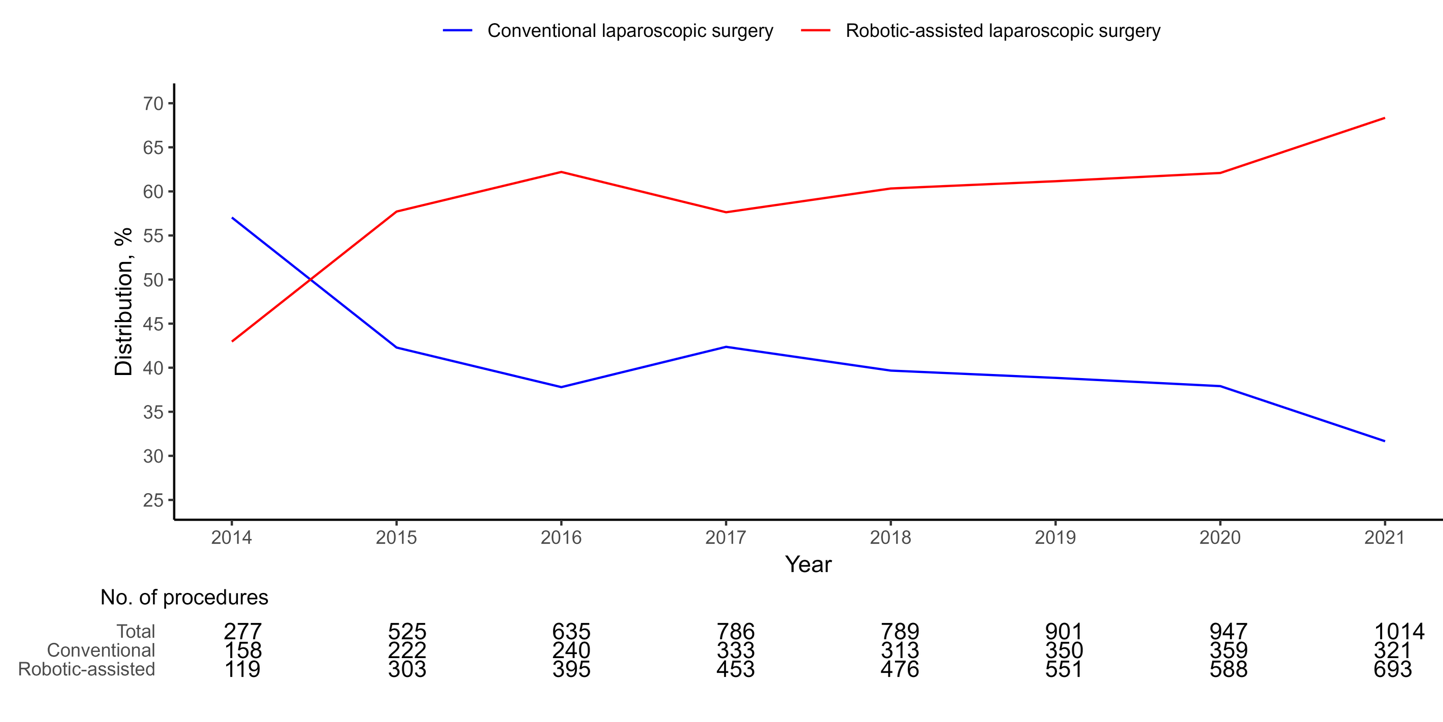
**

**Fig. S1**

Distribution of elective abdominal surgery procedures for rectal cancer of conventional laparoscopic surgery and robotic-assisted laparoscopic surgery per year in Sweden, 2014-2021

**
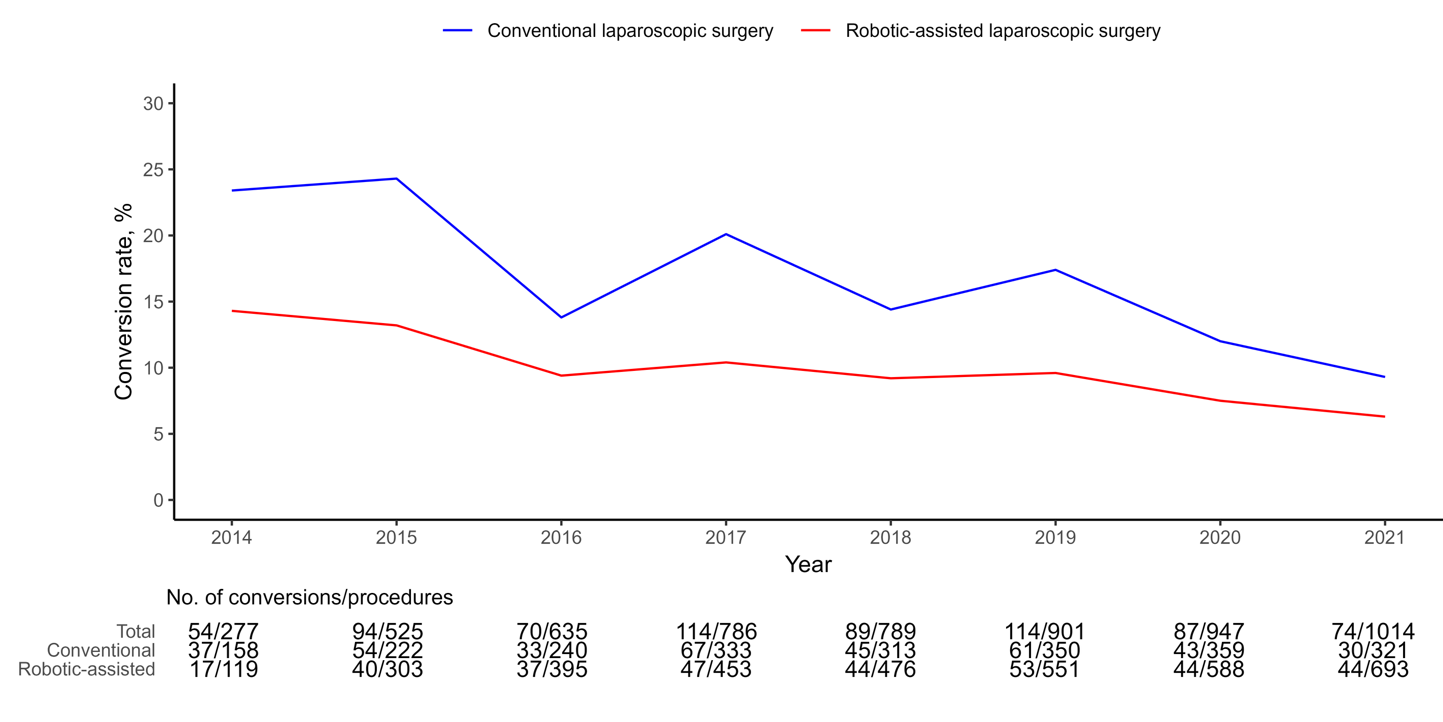
**

**Fig. S2**

Conversion rates in elective abdominal surgery for rectal cancer in conventional laparoscopic surgery and robotic-assisted laparoscopic surgery per year in Sweden, 2014-2021
